# Sea Clutter Reduction and Target Enhancement by Neural Networks in a Marine Radar System

**DOI:** 10.3390/s90301913

**Published:** 2009-03-16

**Authors:** Raúl Vicen-Bueno, Rubén Carrasco-Álvarez, Manuel Rosa-Zurera, José Carlos Nieto-Borge

**Affiliations:** Signal Theory and Communications Department, Superior Politechnic School, University of Alcalá, Alcalá de Henares, 28805, Madrid, Spain

**Keywords:** Neural Networks, Non-linear Signal Processing, Radar, Remote Sensing, Clutter Reduction, Target Enhancement, SCR Improvement

## Abstract

The presence of sea clutter in marine radar signals is sometimes not desired. So, efficient radar signal processing techniques are needed to reduce it. In this way, nonlinear signal processing techniques based on neural networks (NNs) are used in the proposed clutter reduction system. The developed experiments show promising results characterized by different subjective (visual analysis of the processed radar images) and objective (clutter reduction, target enhancement and signal-to-clutter ratio improvement) criteria. Moreover, a deep study of the NN structure is done, where the low computational cost and the high processing speed of the proposed NN structure are emphasized.

## 1. Introduction

The measurement of radar backscatter from an ocean surface, usually referred to as sea clutter, plays an important role in ocean surveillance and remote sensing. In particular, two different points of view may be identified depending on the application. From the first one, sea clutter contains useful information about the ocean surface and the characterization of sea clutter becomes the focal point of the study. From the second one, if the primary objective is the detection of targets, such as ships and/or boats, then, the presence of sea clutter is viewed as a source of interference to be suppressed. The studies presented in this paper are focused on the last case, where the suppression of sea clutter signals and the enhancement of signals related to ship/s play an important role.

The clutter reduction system explained in this paper is proposed to be used in conventional radar systems. These general purpose systems measure only the intensity of the returned electromagnetic echo, where no phase information is given (non-coherent/incoherent radar systems). These kind of systems are commonly used, for instance, in marine traffic control centers. Moreover, the proposed clutter reduction system, among other final applications, can be used to improve the information managed by automatic identification systems (AISs) [1], such as the accurate positioning and tracking of surrounding ships by radar measurements, which lets improve the safety of marine navigation.

Previous experiments of other researchers using different clutter reduction techniques denote that promising results can be achieved. In this way, in [2] a large number of clutter reduction methods applied to different problems are analyzed. This work classifies them both in terms of how they deal with clutter reduction and more importantly, in terms of the benefits and losses depending on the application where they are applied to. Moreover, the same authors of the previous work analyze in [3] an automatic procedure to reduce the level of clutter signals in parallel coordinates plots applied to different problems. The previous referenced work is important in our studies because they work with the same coordinates as the ones used during our experiments.

On the other hand, and focusing on the remote sensing technology we work with in this paper, different approaches have been satisfactorily used. These approaches can be divided, among others, in three categories: conventional methods based on statistical models, image processing-based methods and methods based on neural networks (NNs), which are relatively novel. Before continuing, it is important to note that due to the speed of movement (doppler effect) of the targets (ships) and the sea clutter (waves) considered in our studies, linear filtering cannot be applied (overlapping of the target and clutter spectra). Moreover, the dynamic of the sea clutter is intrinsically nonlinear. So, due to both reasons, a nonlinear signal processing is needed.

In conventional methods, an statistical model is supposed a priori (Weibull-distributed clutter, k-distributed clutter, etc.), which involves the main statistical effects of the clutter. In this way, an approach to reduce ground clutter based on statistical signal processing techniques is proposed in [4]. This approach is based on the use of a simple parametric model, what limits the application of these techniques to other kind of clutters, such as sea clutter. Other approaches, based on statistical signal processing, such as principal component analysis (PCA) [5] and independent component analysis (ICA) [6], have been successfully applied to ground clutter reduction. Moreover, other statistical-based techniques, such as the estimation of target and clutter signal parameters by the use of the likelihood ratio test [7], are used to reduce the level of clutter in surface penetrating impulse radars.

In image processing-based methods, several ones could be cited, but we focus on commonly used methods based on transform domains. One of them is based on the reduction of clutter in radar images by a translation invariant wavelet packet decomposition [8, 9]. Other techniques use the information contained in temporal radar image sequences [10], what makes the system work slow (high computational cost and memory requirements) or with some delay in the presentation of information to the user.

The last category is based on NNs, which is the aim of our proposal. In these methods, the a priori knowledge of the statistical distributions of the target and clutter and their parameters is not necessary, as occur with the conventional (classical) methods. During the NN training, the NN is able to learn the statistical behavior of the training data, what make it a suitable method to infer the statistical distribution of the data (target and clutter) and its parameters. So, for instance, the use of NNs, such as the ones based on radial basis functions (RBFs), denotes the suitable applicability of these techniques to radar target detection based on clutter modeling [11]. Moreover, this kind of NNs are successfully used in final radar applications, such as automatic target detection in clutter by clutter modelling [12], which can be considered as a future application of the clutter reduction system proposed. On the other hand, an Adaptive-Network-based Fuzzy Inference System (ANFIS) architecture has been successfully applied in [13] to classify different kind of weather radar clutters. So, as a NN can be an inner element of an ANFIS architecture, this architecture can be used in the future to improve the clutter reduction achieved by NN-based systems.

After observing that clutter reduction (in general) is successfully applied in different kinds of radar systems (ground, sea, etc.), we now focus on the particular topic presented in this paper, i.e., the reduction of sea clutter in radar images. Moreover, because of the promising results achieved with NNs in previous studies where clutter modeling was used [11, 12], the clutter reduction system proposed in our paper is also based on NNs. But considering that these works [11, 12] are based on RBF-NNs, the proposed system is focused on a different NN category, the multilayer perceptron (MLP), where different activation functions to the RBF-NNs, different NN training algorithm (supervised training) and a different way to select the input data are used. The proposed NN-based sea clutter reduction system also defers from the previous ones based on RBF-NNs because it is proposed as a nonlinear filter instead of a clutter modeling system used to detect radar targets. The proposed system presents several advantages. First, it can be used in commonly used radar systems (incoherent radar systems). Second, due to the used NNs (MLPs) are able to implement nonlinear transfer functions, they are able to solve the problem of separation of clutter and target signals in order to reduce the level of (sea) clutter and enhance the level of target (ship). Third, neither the knowledge of the statistical distribution of the target and clutter nor their parameters are not necessary a priori, as in the conventional/classical methods. In this case, just only a set of radar images and their desired outputs are needed to train the NN. And fourth, the proposed method filters out the sea clutter pixel by pixel in each image and it doesn’t require a sequence of radar images, as several image processing-based techniques do. This advantage makes it faster processing radar images.

In order to cover the explanation of the proposed system, the paper is organized in five sections, including the current one. Section 2. describes the platform used to get the radar measurements, which was successfully used in other research works [10]. The structure and design of the proposed NN-based clutter reduction system is explained in Section 3. The results obtained with the proposed system are presented in Section 4., where a system dimensionality study is carried out and a subjective and an objective analysis of the results is done. So, the results analysis from the subjective point of view focuses on the visual analysis and the subjective interpretation of the radar images at the output of the proposed system. Whereas, the analysis of the results from the objective point of view focuses on two kind of estimations. First, the error between the desired and the obtained radar image outputs is estimated. And second, certain parameters of radar signals are estimated, such as the relationship between the clutter and the target average powers, which let us to estimate the signal-to-clutter ratio (SCR). This performance evaluation let us to estimate the performance improvement achieved by the proposed system. Finally, the main conclusions and contributions of the proposed system are exposed in Section 5., including several aspects that could be improved in the future.

## 2. Measuring and Monitoring Marine System

**Figure 1.**
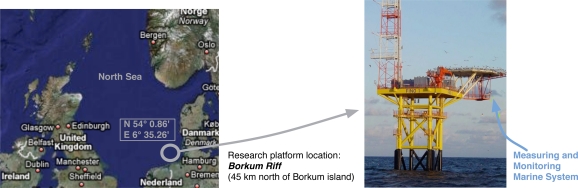
FINO 1 Emplacement in the North Sea and Measuring and Monitoring Marine System Location

The radar measurements considered in our experiments were acquired in the FINO 1 (Forschungsplattformen in Nord-und Ostsee) German research platform located at the German basin of the North Sea (see Figure 1). In this platform, an incoherent radar system is available and a WaMoS II system [14] is installed, which compose the measuring and monitoring marine system used during the experiments (Figure 1). The WaMoS II system is an operational Wave Monitoring System developed by the German research institute GKSS and commercialized by OceanWaveS GmbH. This system acquires every five minutes a temporal sequence of 32 consecutive radar images. The intensity of each radar image cell is coded without sign and 1 byte (8 bits), what sets a dynamic range of [0 − 255]. The sampling time (*T_s_*) of this temporal sequence of radar images corresponds to the antenna rotation period. The spatial resolutions (*R_x_* and *R_y_*) of each image depends on the azimuthal and range resolutions of the radar system.

**Figure 2.**
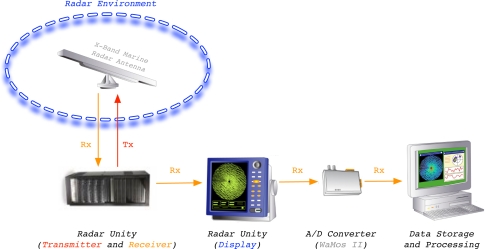
Measuring and Monitoring Marine System

The measuring and monitoring system used during the experiments is shown in Figure 2. This system is a standard marine X-band radar, which works on HH polarization, has an incoherent logarithmic amplier and no frequency agility. Once the radar data are acquired and plotted in the radar unity display, its analog video signal is digitized by the Analog-to-Digital (A/D) converter WaMoS II [15]. The digitized images are formatted in order to be processed by a standard computer to perform the desired ending radar application by software. In this case, the radar application we present in this paper is related to sea clutter reduction. This reduction tries to emphasize the information contained in a radar image related to the desired target/s (ship/s) and reduce the information related to the clutter. Because of the radar raw data from WaMoS II measurements are used, no signal preprocessing is done before the proposed clutter reduction system is applied.

The technical specifications of the used radar measurement system are given in table 1. It is important to note that this system is highly configurable according to the configuration of some of its technical parameters. Moreover, the inhibition of several scan sectors during the radar measurements is possible, as can be observed in the radar images presented in Section 4.

## 3. NN-based Clutter Reduction System

NNs, and exactly MLPs, are able to learn the statistical distribution of the input data to give the desired output from the input-output data relationship during a training process. In this way, considering a supervised training, where the desired outputs are known, the input data are presented to the NN and their corresponding outputs are obtained. According to the obtained output, the NN is able to adapt its transfer function during the training process (design stage) in order to minimize a certain error between the desired and obtained outputs. Once the NN is trained (design stage), it is tested (test stage) in order to analyze its behavior with other input data, which are different of the data used during the design stage. It is important to note that in the test stage is not mandatory to know which are the desired outputs as occur in the design stage. So, due to the above mentioned capabilities of learning from the input-output data relationship of an MLP during a supervised training, an MLP is used to learn the statistical behavior of the clutter and targets (ships) signals contained in radar images. The different kinds of clutter and target are exposed in the presentation of the radar image database used during the experiments (see subsection 4.1.). This learning lets the MLP maximizes the separation between target and clutter information at its output. This separation is evaluated in the results section as the SCR improvement, where, in this case, the desired output radar images are known in order to give an objective performance of the system. On the other hand, the system, once designed, can work with this knowledge of the desired output, because a subjective analysis can be also done, as it is shown in the results section too.

Once the basis of the proposed NN-based clutter reduction system is explained, several aspects are described in the next subsections. These aspects focus on how the radar image at the output of the system is achieved, how the output error is estimated, what kind of MLP training algorithm is used, how to design and test the system, what kind of evaluation of the system performance is done and which are the steps followed to study the dimensionality of the NN.

### 3.1. System Processing: Output Radar Image Achievement

**Figure 3.**
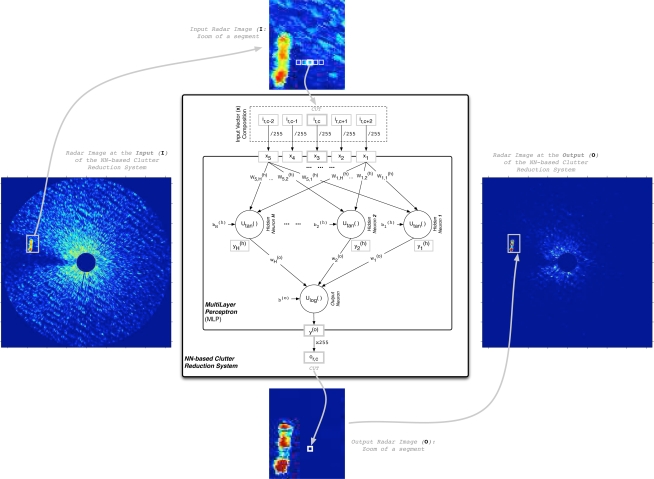
NN-based clutter reduction system

Independently of the MLP stage (design or test), the way the NN-based clutter reduction system processes the data of the radar measurements included in the input radar image (I) to obtain the processed output radar image (O) is summarized in Figure 3. This figure shows how the MLP output *y*^(o)^ is obtained for a cell under test (CUT). Moreover, it is also shown how this output is assigned to the corresponding cell of the output radar image (*o_r,c_*), which is established by the *r*-th row and *c*-th column of the CUT in the input radar image. Note that only one MLP output is selected because the objective is processing each valid cell of the input radar image and obtaining an output radar image of the same size as the input one. In our case of study, a total of 5 radar return measurements for different positions (range and azimuth) are considered, where they are selected from horizontal radar image raw data. Note that due to the circular symmetry of the coverage and the freedom of waves movement and ships navigation (they can move in whatever direction), a vertical orientation could be selected obtaining similar system performances. Other choice could be use a different shape of selecting the surrounding data of the CUT, what could be studied in the future. Moreover, note that from these 5 selected measurements of **I** for each CUT, the central element (*i_r,c_*) corresponds to the CUT, and the others correspond to surrounding measurements, where a symmetric distribution of the selected data is done. This symmetric selection is done in order to have the same quantity of information from both sides. This number of measurements (empirically obtained) is selected as a trade-off between the system performance, the MLP computational complexity and the size of the targets (ships) under study. Note that 5 cells with a range resolution of 7.5 m (see table 1) involve a distance of 37.5 m, which is the minimum range necessary to englobe the beam of all the ships under study (see table 2 of subsection 4.1.) in case they are perpendicularly placed with respect to the orientation of the selection of input data. As can be observed, the MLP architecture depends on the characteristics of the target to be enhanced. On the other hand, following the nomenclature established in Figure 3 and taking into account the dynamic range of the input data (8 bits: dynamic range of [0 − 255]), the processed output (*o_r,c_* = round(*y*^(o)^ *·* 255)) for a given CUT and its surrounding cells, which are contained in the input vector **x** = [*x*_1_, *x*_2_, *x*_3_, *x*_4_, *x*_5_] = [*i_r,c−_*_2_, *i_r,c−_*_1_, *i_r,c_, i_r,c_*_+1_*, i_r,c_*_+2_]/255, is given by:

(1) $$y^{\left(o\right)}=f_{\text{NN}}(x)$$

where *f*_NN_(*·*) denotes the transfer function implemented by the NN. Note that the NN inputs (**x**) are normalized to a range [0 − 1]. Moreover, due to the activation function selected for the NN output neuron, its output (*y*^(o)^) is limited to the range [0 − 1], as shown below.

Once the way the radar measurements selection to the MLP input for each CUT is exposed and justified, the MLP output computation is presented. In our case of study, we have considered an MLP composed of 5 inputs, *H* hidden neurons and 1 output neuron, i.e., a structure 5/*H*/1 with two layers (one hidden layer and the output layer), where *H* is a parameter under study in our experiments. Moreover, due to inner property of the unknown nonlinear dynamic of the sea clutter during time and the possible overlapping of the moving target and clutter spectra (doppler effect), a nonlinear system is needed because linear solutions are not suitable for this purpose. In this way, this nonlinear function can be achieved by the used NN and its implemented transfer function (*f*_NN_(*·*)).

The way to obtain the MLP output is presented below in the same way the MLP processes the data, i.e., first the hidden neuron outputs are achieved and finally the MLP output is obtained. In this way, considering $v_{j}^{\left(h\right)}$ is the overall weighted input of the *j*-th hidden neuron, which is obtained by eq. (2), the *j*-th hidden neuron output ( $y_{j}^{\left(h\right)}$) can be computed by eq. (3), where a (nonlinear) *hyperbolic tangent* activation function (*ψ*_tan_(*·*)) [16] is used.

(2) $$v_{j}^{\left(h\right)}=\left(\sum_{i=1}^{5}x_{i}\cdot w_{i,j}^{\left(h\right)}\right)+b_{j}^{\left(h\right)}$$

(3) $$y_{j}^{\left(h\right)}=\psi_{\text{tan}}\;\left(v_{j}^{\left(h\right)}\right)=\frac{\text{sinh}\left(v_{j}^{\left(h\right)}\right)}{\text{cosh}\left(v_{j}^{\left(h\right)}\right)}=\frac{e^{v_{j}^{\left(h\right)}}-e^{-v_{j}^{\left(h\right)}}}{e^{v_{j}^{\left(h\right)}}+e^{-v_{j}^{\left(h\right)}}}$$

Note that the element $w_{i,j}^{\left(h\right)}$ denotes the MLP synaptic weight that connects the *i*-th input (*x_i_*) with the *j*-th hidden neuron (see Figure 3). So, **W**^(h)^ is the matrix that contains the synaptic weights that connect the MLP inputs with the MLP hidden neurons. Moreover, $b_{j}^{\left(h\right)}$ denotes the bias of the *j*-th hidden neuron, where the row vector **b**^(h)^ contains all the hidden neuron biases. For our case of study, **W**^(h)^ contains a total of [5×*H*] synaptic weights and **b**^(h)^ contains a total of [1×*H*] bias weights.

Following the MLP computation procedure, and considering *v*^(o)^ is the overall weighted input of the output neuron, which is obtained by eq. (4), the neuron output (*y*^(o)^) can be computed by eq. (5). In this case, this neuron uses a (nonlinear) *logistic* activation function (*ψ*_log_(·)) [16] because of the need that the MLP output is limited between 0 and 1.

(4) $$v^{\left(o\right)}=\left(\sum_{j=1}^{H}y_{j}^{\left(h\right)}\cdot w_{j}^{\left(o\right)}\right)+b^{\left(o\right)}$$

(5) $$y^{\left(o\right)}=\psi_{\text{log}}\;\left(v^{\left(o\right)}\right)=\frac{1}{1+e^{v(o)}}$$

Note that the element $w_{j}^{\left(o\right)}$ denotes the MLP synaptic weight that connects the output of the *j*-th hidden neuron ( $y_{j}^{\left(h\right)}$) with the output neuron (see Figure 3), where the column vector **w**^(o)^ contains all these weights. Moreover, *b*^(o)^ denotes the bias of the output neuron. For our case of study, **w**^(o)^ contains a total of [*H*×1] synaptic weights and *b*^(o)^ contains a total of [1×1] bias weight.

Summarizing, the NN-based clutter reduction system output given by eq. (1) can be computed in four steps following the eq. (2)–(5). But, using a matrix notation, all this procedure can be summarized in two steps, which are given by eq. (6) and (7).

(6) $$y^{\left(h\right)}=\psi_{\text{tan}}\;\left(x\cdot W^{\left(h\right)}+b^{\left(h\right)}\right)$$

(7) $$y^{\left(o\right)}=\psi_{\text{log}}\;\left(y^{\left(\text{h}\right)}\cdot w^{\left(o\right)}+b^{\left(o\right)}\right)$$

### 3.2. Output Error Estimation

Once the MLP output is obtained by eq. (6) and (7) for a certain CUT of a given radar image, it is important to estimate how good is the result achieved by the MLP. For this purpose, the measurement of a certain error is needed. So, the difference between the desired (*d*^(o)^) and achieved (*y*^(o)^) MLP outputs is computed by eq. (8).

(8) $$e=d^{\left(o\right)}-y^{\left(o\right)}$$

If this error is calculated for all the valid pixels of all the radar images included in a given set of radar images, the error can be computed in our case of study as:

(9) $$e_{m}=\frac{d_{r,c,k}}{255}-\frac{o_{r,c,k}}{255},\;\;\;m=1,2\ldots P$$

where *d_r,c,k_* and *o_r,c,k_* denote the elements placed at the *r*-th row and *c*-th column of the *k*-th desired (D) and obtained (O) MLP output images of the set, respectively. Note that both elements are normalized by a constant of $\frac{1}{255}$ (inverse of the dynamic range of the radar image cells/pixels) because the range of the MLP output varies from 0 to 1. Moreover, the index *m* depends on the indexes *r*, *c* and *k*, which is calculated as *m* = *r* + (*c −* 1)*N* + (*k −* 1)*NN*. For our case of study, the indexes *r* and *c* varies from 1 to *N*, which denotes that the radar images of the set are squared and have the same size (*N*×*N* pixels), and the index *k* varies from 1 to *M*, where *M* is the number of radar images of the set. Finally, the index *m* varies from 1 to *P*, where *P* depends on the values of *N* and *M* in the following way: *P* = *NNM*.

Finally, the error function selected for our studies is the mean squares (MS) error [16], which is computed by eq. (10) in a general case or by eq. (11) for our case of study.

(10) $$e_{\text{MS}}=\frac{1}{P}\sum_{m=1}^{P}\frac{1}{2}e_{m}^{2}$$

(11) $$e_{\mathrm{MS}}=\frac{1}{M}\sum_{k=1}^{M}\left(\frac{1}{N}\sum_{r=1}^{N}\left(\frac{1}{N}\sum_{c=1}^{N}\frac{1}{2}{\left(\frac{d_{r,c,k}}{255}-\frac{O_{r,c,k}}{255}\right)}^{2}\right)\right)$$

The MS error computation for a given set of radar images exposed above has a fixed value when the synaptic weights and biases of the MLP does not change in time (the obtained output is the same). But, during the MLP training (NN-based clutter reduction system design stage), these weights and biases change (the obtained output changes). In this way, different errors are obtained in each iteration *n* of the training algorithm. So, the MS error computed at the *n*-th algorithm iteration is given by

(12) $$e_{\text{MS}}[n]=\frac{1}{M}\sum_{k=1}^{M}\left(\frac{1}{N}\sum_{r=1}^{N}\left(\frac{1}{N}\sum_{c=1}^{N}\frac{1}{2}{\left(\frac{d_{r,c,k}}{255}-\frac{O_{r,c,k}[n]}{255}\right)}^{2}\right)\right)$$

where it is important to note that the *m*-th MLP output $\left(o_{r,c,k}[n]=255\cdot y_{m}^{\left(o\right)}[n]\right)$ is the only parameter that changes in this expression because it depends on the nonlinear transfer function implemented by the MLP (see eq. (2)–(5)), which depends on the values of the MLP weights and biases at the *n*-th iteration.

### 3.3. Training Algorithm

Taking into account this dependency of the MS error with respect to the algorithm iteration, the MLP weights and biases are updated to minimize the MS error in the following iteration (*e*_MS_[*n* + 1]). Several training algorithms could be used to minimize this error. In this way, second order optimization techniques could be used, as the ones based on Newton, Quasi-Newton and Levenberg-Marquardt methods [16]. The Newton method is characterized because of the Hessian matrix is estimated by the approximation of second order derivates. Whereas, the Quasi-Newton method approximates the Hessian matrix by the Jacobian matrix, where first order derivatives must be approximated. Finally, the Levenberg-Marquardt method needs to estimate the Jacobian matrix, where first order derivatives must be approximated. All these methods present two main advantages: great speed of convergence and high success rate in finding the global minima. Nevertheless, they need a huge number of training observation vectors (data) to estimate the Hessian or Jacobian matrixes with a minimum accuracy. Moreover, this training is effective in terms of computational cost if low size MLPs are used because of, considering that the MLP is composed of *F* weights, both estimated matrixes are of sizes *F*×*F*. So, if *F* increases linearly, the computational cost needed in each algorithm iteration increases exponentially. Therefore, as we don’t know a priori which is the optimum MLP size in our case of study, the learning algorithm used during the MLP design is the error *back-propagation* algorithm with variable learning rate (α) and momentum (*μ*) [17]. This training algorithm doesn’t need so much computational cost, as the previously exposed approaches, especially for big MLP sizes. This algorithm lets update the MLP synaptic weights that connect the inputs with the hidden neurons (**W**^(h)^) and those that connect the hidden neurons with the output neuron (**w**^(o)^) according to eq. (13) and (14), respectively.

(13) $$w_{i,j}^{\left(h\right)}[n+1]=w_{i,j}^{\left(h\right)}[n]+\alpha [n]\cdot \frac{1}{P}\cdot \sum_{m=1}^{P}\left(\delta_{m,j}^{\left(h\right)}[n]\cdot x_{m,i}\right)+\mu \cdot w_{i,j}^{\left(h\right)}[n-1],\{\begin{array}{l} i=1,2\ldots 5\\ j=1,2\ldots H \end{array}$$

(14) $$w_{j}^{\left(o\right)}[n+1]=w_{j}^{\left(o\right)}[n]+\alpha [n]\cdot \frac{1}{P}\cdot \sum_{m=1}^{P}\left(\delta_{m}^{\left(o\right)}[n]\cdot y_{m,j}^{\left(h\right)}[n]\right)+\mu \cdot w_{j}^{\left(o\right)}[n-1],\;\;j=1,2\ldots H$$

Moreover, the biases of the hidden (**b**^(h)^) and output (*b*^(o)^) neurons are updated by eq. (15) and (16), respectively. Note that these expressions are very similar to the synaptic weight updates but considering that the virtual input of the neuron connected by the bias is unity, i.e., *x*_*m*,0_[*n*] = 1 and $y_{m,0}^{\left(o\right)}[n]=1$.

(15) $$b_{j}^{\left(h\right)}[n+1]=b_{j}^{\left(h\right)}[n]+\alpha [n]\cdot \frac{1}{P}\cdot \sum_{m=1}^{P}\left(\delta_{m,j}^{\left(h\right)}[n]\cdot 1\right)+\mu \cdot b_{j}^{\left(h\right)}[n-1],\{\begin{array}{l} i=1,2\ldots 5\\ j=1,2\ldots H \end{array}$$

(16) $$b^{\left(o\right)}[n+1]=b^{\left(o\right)}[n]+\alpha [n]\cdot \frac{1}{P}\cdot \sum_{m=1}^{P}\left(\delta_{m}^{\left(o\right)}[n]\cdot 1\right)+\mu \cdot b^{\left(o\right)}[n-1],\;\;j=1,2\ldots H$$

Note that all the training parameters and inner signals of the MLP depends on *n*, except the momentum constant (*μ*) and the input vector (**x***_m_*), where *m* indicates the index of the CUT, as exposed previously. Moreover, it is important to clear that the matrix δ^(h)^[*n*] and the vector δ^(o)^[*n*] are the local derivatives of the MS error function at the *n*-th iteration of the algorithm with respect to its corresponding neuron output, i.e., this partial derivative is a way to estimate the sensibility of the neuron weights with respect to the error. Both matrix and vector can be achieved by eq. (17) and (18), respectively [17]. Note that the MLP is trained in a batch mode, i.e., the weights and biases are not updated until the error for all the *P* cells of all the radar images of a given set are obtained.

(17) $$\delta_{m,j}^{\left(h\right)}[n]=\psi_{\text{tan}}^{\prime }\;\left(v_{m,j}^{\left(h\right)}[n]\right)\cdot \left[\left(\sum_{i=1}^{H}\delta_{m}^{\left(o\right)}[n]\cdot w_{i}^{\left(o\right)}[n]\right)+\delta_{m}^{\left(o\right)}[n]\cdot b^{\left(o\right)}[n]\right],\{\begin{array}{l} j=1,2\ldots H\\ m=1,2\ldots P \end{array}$$

(18) $$\delta_{m}^{\left(o\right)}[n]=\psi_{\text{log}}^{\prime }\;\left(v_{m}^{\left(o\right)}[n]\right)\cdot e_{m}[n],\;\;m=1,2\ldots P$$

In eq. (17) and (18), *ψ^′^*_tan_(*·*) and *ψ^′^*_log_(*·*) denote the partial derivatives of the activation functions *ψ*_tan_(*·*) and *ψ*_log_(*·*), respectively, with respect to the overall weighted inputs $v_{m,j}^{\left(h\right)}[n]$ and $v_{m}^{\left(o\right)}[n]$, respectively. After applying these partial derivatives to eq. (3) and (5), eq. (19) and (20) are achieved, respectively, where it is important to remember that $y_{m}^{\left(o\right)}[n]=o_{r,c,k}[n]/255$.

(19) $$\psi_{\text{tan}}^{\prime }\;\left(v_{m,j}^{\left(h\right)}[n]\right)=\left[1-y_{m,j}^{\left(h\right)}[n]\right]\cdot \left[1+y_{m,j}^{\left(h\right)}[n]\right],\{\begin{array}{l} j=1,2\ldots H\\ m=1,2\ldots P \end{array}$$

(20) $$\psi_{\text{log}}^{\prime }\;\left(v_{m}^{\left(o\right)}[n]\right)=\left[1-y_{m}^{\left(o\right)}[n]\right]\cdot y_{m}^{\left(o\right)}[n],\;m=1,2\ldots P$$

Once the partial derivatives have been computed, and the weights are updated according to the actual parameters of the algorithm (*α*[*n*] and *μ*), the learning rate is automatically adapted for the following algorithm iteration (*α*[*n* + 1]) by

(21) $$\alpha [n+1]=\{\begin{array}{ll} \alpha [n]*(1+\alpha_{\text{inc}}) & \text{if}\;e_{\text{MS}}[n]<e_{\text{MS}}[n-1]\\ \alpha [n]*(1-\alpha_{\text{dec}}) & \text{if}\;e_{\text{MS}}[n]\geq e_{\text{MS}}[n-1]*(1+p_{\text{max}})\\ \alpha [n] & \text{otherwise} \end{array}$$

where the parameters *α*_inc_ and *α*_dec_ are the increasing and decreasing rates of the learning rate (*α*). Moreover, in order to warranty the stability of the learning algorithm, a learning rate constraint is set. This constraint controls the maximum error increase in order not to surpass a certain limit. In this way, this constraint is controlled by the parameter *p*_max_.

Finally, it is important to note that due to the specialization of the MLP during its training in the design stage, a new set of radar images (*validation set*) is used to externally validate the training process and to stop it before the MLP is memorizing the training set. This external validation decreases the specialization of the MLP after training (memorization of the radar images contained in the *training set*) and increases its generalization when new radar images (*test set*) are presented at its input.

### 3.4. Design and Test Stages

Previously, the way the system output obtaining (processed radar image) and its training algorithm are exposed. But, how can we design the system?, i.e., how can we make that the system learn what we want? In this way, first the desired output of the system must be set, and after the MLP is trained (a part of the system design) in a supervised way, what is exposed below.

Next, the procedure to obtain the desired outputs (radar images) is exposed. The desierd output image will be a binary image, where a cell of this image with a value of 1 indicates that a target is present there, whereas a value of 0 indicates that target is absent (clutter). The desired outputs are only necessary to design the system (learning if target is present or not in a cell), because, once it is already designed, it will work without any knowledge related to whether a target (ship) is present in the radar scene (in a cell) or not. Nevertheless, we also create the desired outputs of the system in the test stage in order to give an objective measurement of its performance, remembering that this step will not be done when the proposed system is working at steady state. The procedure to obtain the desired radar images at the output of the system in our experiments is:

- First: We take the 32 radar images of a sequence that contains a target (a ship). For this sequence, a statistical study of its length and beam is done, considering for this study the zones of the image where the target is located.
- Second: A model of the target is done according to the mean values of length and beam, rounding the edges of this model in order to approximate to the real shape of a ship.
- Third: The ship model obtained for the sequence under study is manually superimposed in each radar image until the model is correctly placed over the ship in the radar image. It is important to note that the ship is continuously changing its relative position to the radar emplacement in both angle and range, what is also considered in this procedure. In this step, it is necessary to be careful with the electromagnetic shadows that produce some ships of huge volume.

Once the desired outputs are determined and the training algorithm is known, the system can be now designed. In this way, the procedure followed to design the NN that compose the NN-based clutter reduction system is:

- First: The NN structure is created, in a general case, with 5 inputs, *H* hidden neurons and 1 output neuron (structure 5*/H/*1).
- Second: Once the NN is created, it is initialized using the Nguyen-Widrow algorithm [18]. This initialization algorithm lets the training algorithm to increase its speed of convergence and to find a minimum of the error surface at the end of the training with high success rate. But it does not warranty that the achieved minimum is always the lowest one (the global minimum). This high success rate of finding a local or global minima at the end of the training is due to the NN weights are initialized considering aspects of the training data (ship and sea clutter) such as the mean, maximum and minimum values, which lets to start the training with some knowledge of the data.
- Third: The initial value of the learning rate in the first algorithm iteration (*n* = 0) is set to α[0] = 0.05, which is evolving during the training algorithm progress by eq. (21). Moreover, the incremental and decremental rates of the learning rate are set to *α*_inc_ = 0.05 and *α*_dec_ = 0.25, respectively, and the maximum error increase from one iteration to the next one is set to *p*_max_ = 0.04.
- Fourth: The momentum constant is set to *μ* = 0.9, which warranties a certain stability in the training algorithm.
- Fifth: The maximum number of algorithm iterations during the NN design (training with external validation) is set to 200. Nevertheless, the NN training is usually stopped due to the loss of generalization. This loss of generalization is estimated by the MS error in the validation set. So, the training algorithm stop is produced when the MS error calculated for the validation set increases during the following algorithm iterations. This MS error increase indicates that the NN is specializing (memorizing) in the radar images of the training set and loosing generalization capabilities to extrapolate this acquired knowledge to other radar images, as those of the validation set.
- Finally, mention that the NN training described in the previous steps is repeated ten times due to the achievement of the lowest minimum error is not always warranted for one execution of the training algorithm. After that, the best trained NN is selected in terms of the maximum average SCR improvement achieved in the validation set (external validation). The used SCR improvement is estimated by the difference between the SCR at the output of the proposed system and the SCR at its input. Note that the SCR is calculated by the decimal logarithmic relationship between the powers of the signal where the target is present and where it is absent (only clutter).

The stability of the training algorithm is warranted for the selected parameters of the algorithm [17]. On the other hand, its convergence to a minimum of the error surface is possible because of the followed training procedure. So, once the system is designed, it is necessary to test its performance. In this way, a new set of radar images, never seen during the design stage, is presented to the system. For this new set, several subjective and objective parameters can be obtained, which are exposed in the next subsection.

### 3.5. Performance Evaluation

Both in the design and test stages previously exposed, the output radar images can be analyzed in terms of subjective interpretation comparing them with those at the input of the system. But, due to the personal interpretation of these results, several objective measurements of the performance are used. Because this performance evaluation is used to give an objective measurement of its performance, it is necessary to know which are the desired radar images at the output of the system. But note that once the system is designed and it is working at steady state, this performance evaluation can not be done because it is difficult to a priori know where the radar target is exactly placed, unless a detector is applied. The objective parameters used in our experiments are:

- For a given radar image:
  – The MS error computed by eq. (11) when *M* = 1, where the obtained/processed radar image and the desired radar image are considered.
  – The clutter power (*P*_c_) improvement (reduction for a negative value) is obtained as the difference between the clutter powers (in dBm) at the output and input of the system. The estimations of these powers are obtained from the zones of the radar image where target (ship) is absent.
  – The target power (*P*_t_) improvement (enhancement for a positive value) is obtained as the difference between the target powers (in dBm) at the output and input of the system. The estimations of these powers are obtained from the zones of the radar image where target (ship) is present.
  – The signal-to-clutter ratio (SCR) improvement is obtained as:
    (22) $${\text{SCR}}^{\text{imp}}(\text{dB})={\text{SCR}}^{\text{out}}(\text{dB})-{\text{SCR}}^{\text{in}}(\text{dB})$$
    where the SCRs at the input and output of the system are obtained by eq. (23) and (24), respectively.
    (23) $${\text{SCR}}^{\text{in}}(\text{dB})=P_{t}^{\text{in}}(\text{dBm})-P_{c}^{\text{in}}(\text{dBm})$$
    (24) $${\text{SCR}}^{\text{out}}(\text{dB})=P_{t}^{\text{out}}(\text{dBm})-P_{c}^{\text{out}}(\text{dBm})$$
- For a given set of radar images:
  – The average *P*_c_ improvement (reduction for a negative value) is obtained as the difference between the clutter powers (in dBm) at the output and input of the system for all the cells of the radar images of the set where target (ship) is absent. Note that this estimation can be different of the mean value of the *P*_c_ improvement achieved for each radar image because the number of cells related to target absent can be different from one image to other. Take as an example the case where the target is not completely inside the radar coverage in one image and in the next radar image of the sequence it is completely inside.
  – The average *P*_t_ improvement (enhancement for a positive value) is obtained as the difference between the clutter powers (in dBm) at the output and input of the system for all the cells of the radar images of the set where target (ship) is present. Note that this estimation can be different of the mean value of the *P*_t_ improvement achieved for each radar image because the number of cells related to target presence can be different from one image to other. Take as an example the same as previously.
  – The average SCR improvement is obtained as the mean value of the SCR improvement achieved for each of the *M* radar images of the set, i.e.:
    (25) $${\text{SCR}}_{\text{av}}^{\text{imp}}(\text{dB})=\frac{1}{M}\sum_{i=1}^{M}{\text{SCR}}_{i}^{\text{out}}(\text{dB})-{\text{SCR}}_{i}^{\text{in}}(\text{dB})$$

### 3.6. Dimensionality

The procedure followed to make a study of the dimensionality of the proposed system is based on the design stage and the performance evaluation of the system exposed previously. So, this dimensionality study tries to find which is the best NN size (5*/H/*1) in terms of objective parameters (clutter reduction and target enhancement, what involves an SCR improvement). In this way, this procedure is based on:

- The training, validation and test sets are always the same for all the experiments done for different NN sizes.
- Ten different NNs are initialized for each size (5*/H/*1) considered in our studies using the Nguyen-Widrow algorithm [18]. As mentioned in the design stage description (see subsection 3.4.), this initialization algorithm helps the NN training algorithm to start from a point in the error surface that leads it to find a local or global minima of the error surface at the end of the training.
- Each NN is trained by the error *back-propagation* algorithm with variable learning rate and momentum, where an external validation of the training progress is done. This validation tries to stop the training before the NN is specializing or memorizing the training set and, in consequence, loosing generalization capabilities. The same algorithm parameters as the ones used in the design stage (see subsection 3.4.) are used.
- Once the ten NNs are created and trained for a given size (5*/H/*1), the best NN of them is selected. The selection is done according to the maximum average SCR improvement considering the radar images of the validation set.
- Finally, this procedure is repeated for each NN size (5*/H/*1) we want to study, where the best NN size is selected in terms of the maximum average SCR improvement achieved considering the radar images of the validation set.

## 4. Results

The current section presents the results obtained with the NN-based clutter reduction system proposed in section 3. for the radar images obtained by the radar system described in Section 2. So, first, the database of radar images used during the experiments is presented in subsection 4.1., where the kinds of clutter and target (ship) considered in the study are exposed. After, a study of the dimensionality of the NN structure is given in subsection 4.2. in order to propose a NN size to work with. And finally, several radar images processed by the proposed NN-based clutter reduction system are shown in subsection 4.3., where a subjective analysis of them is done. On the other hand, the same radar images are analyzed from an objective point of view in subsection 4.4.

### 4.1. Radar Image Database: Training, Validation and Test Sets Compositions

The database selected for the experiments is composed of 12 different radar data image sequences obtained by the radar measurement system presented in Section 2. All of them are different each other in order to cover different sea states (height, period, and character of waves on the surface of a large body of water) [19, 20] among sea states 1–5 proposed by the World Meteorological Organization (WMO) [21]. Moreover, one half (6 seq.) of the sequences considered in our study corresponds to only clutter conditions (target is absent). The other half (6 seq.) corresponds to situations where target (ship) is present in the clutter-governed environment. Table 2 contains the kinds of target (ship) considered in our studies and their sizes. So, this variety of sequences with different radar environments (sea states and ships) tries to cover the different possibilities where the radar can work. In this way, the first 8 radar image sequences of these 12 sequences are dedicated to design the NN-based clutter reduction system, where 4 of them (2 with target and clutter and 2 with only clutter) are dedicated to compose the training set and the remaining 4 sequences (2 with target and clutter and 2 with only clutter) are used to compose the validation set. On the other hand, the other 4 sequences (2 with target and clutter and 2 with only clutter) of the 12 sequences of the database are dedicated to test the system performance.

**Figure 4.**
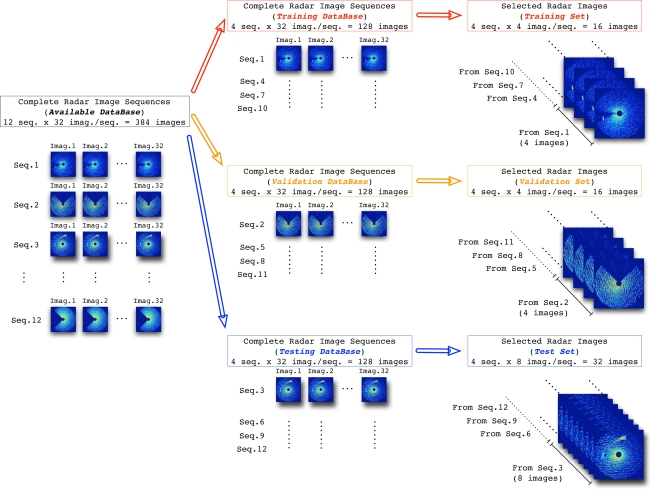
Training, Validation and Test Sets Composition from the Radar Image Database

It is important to note that each image, whose size is *N*×*N* pixels, contains a total of 332*,* 929 pixels (*N* = 577). But, a maximum of 257*,* 307 pixels are valid for our studies (the parts outside the radar coverage are removed from the image). Moreover, when the radar coverage is lower than 360°, i.e., a coverage area is rejected of the radar coverage, the number of valid pixels of the radar image of a given sequence is lower than this maximum. In order to compose the final sets used in the design and test stages, a few radar images concerning the most representative ones of each sequence are selected. In this way, 4 radar images are selected from each sequence considered in the training and validation databases, whereas 8 radar images are selected from each sequence of the testing database. This image selection is done not only to train the NN in a reasonable time and with a warranty to obtain good NN-based clutter reduction system performances, but also to avoid the NN specialization in the training and validation sets. In this way, the different composition of the sets used during the experiments is summarized in Figure 4, where their sizes are also depicted.

### 4.2. NN-based Clutter Reduction System: Dimensionality

Following the procedure exposed in subsection 3.6., a study of the best NN size with a structure 5*/H/*1 is done. In this way, 11 representative sizes are selected for this system dimensionality study. The sizes consider the following number of hidden neurons (*H*): 4, 6, 8, 10, 12, 14, 16, 18, 20, 25 and 30.

The results obtained for the best NNs of the 11 different sizes considered in the study are given in table 3. This table includes the average *P*_c_, *P*_t_ and SCR improvements achieved for the training, validation and test sets. Analyzing these results, several aspects can be remarked:

**First:** The achieved average SCR improvements, clutter reductions (negative improvements of the *P*_c_) and target enhancements (positive improvements of the *P*_t_) are always better under design conditions (training and validation sets) than under test conditions (test set). This is due to the NN is learning the statistical conditions of the radar environments presented in the training and validation sets. But note that the achieved improvements are similar for both design and test conditions, specially for NNs of low size. This is due to, on one hand, the NN is maintaining the generalization capabilities with low sizes and not with high sizes. On the other hand, due to the correct selection of the radar images that contains the training and validation sets, the performance generalization of the NN to other radar images never seen before, as the ones that compose the test set, is higher.
**Second:** The average SCR improvement is not the same as the difference between the average *P*_t_ and *P*_c_ improvements. It happens because of the average *P*_c_ improvement is obtained as the difference between the clutter powers (in dBm) at the output and input of the system for all the cells of the radar images of the set where target (ship) is absent. Whereas this estimation can be different of the mean value of the *P*_c_ improvement achieved for each radar image because the number of cells related to target absent can be different from one image to other. The same occurs with the average *P*_t_ but when the target is present. To appreciate it, consider the case where the target is not completely inside the radar coverage in one image and in the next one it is already inside.
**Third:** As can be observed in the achieved average *P*_c_ improvements (clutter reduction), the optimum NN size needs to include 6 hidden neurons in its hidden layer (5*/*6*/*1) because greater or lower NN sizes achieve lower performances. This is due to the NN only needs a few number ((5 *·* 6 + 1 *·* 6) + (6 *·* 1 + 1 *·* 1) = 43) of adaptive parameters (synaptic weights (**W**^(h)^ and **w**^(o)^) and biases (**b**^(h)^ and *b*^(o)^) of the NN) to find a good solution. Moreover, it is observed that having more than 43 adaptive parameters (weights and biases) in the NN-based clutter reduction system is not efficient because both the computational cost and the memory requirements to store the parameters increase, and the most important aspect, its performance decreases. This performance decrease, due to the NN size increase, is based on the specialization that acquires the NN after training and its corresponding loss of generalization [22, 23] to find a good solution for other radar images never seen before.
**Fourth:** The average *P*_t_ improvement (target enhancement) is very similar in the training and validation sets for all the cases under study (NN size) and slightly different in the test set when the NN size increases (loss of generalization).
**Fifth:** As a general conclusion related with the previous effects, the average SCR improvement is maximum for a size of 6 hidden neurons in its hidden layer (structure 5*/*6*/*1). Moreover, less or more hidden neurons provokes a decrease in its performance, where the higher is the number of hidden neurons, the greater is the performance decrease. This performance decrease with the NN size increase is due to the specialization the NN is acquiring, i.e., the loss of generalization capabilities.

### 4.3. NN-based Clutter Reduction System: Subjective Analysis

In order to characterize the performance of the proposed NN-based clutter reduction system, first it is necessary design it setting the training algorithm parameters and the NN structure (NN size), and finally test the designed system. In this way, the best NN size is 5*/*6*/*1, as obtained from the dimensionality study. The NN training algorithm used during the design stage, i.e., the *back-propagation* algorithm with variable learning rate and momentum (see subsection 3.3.), is based on several configurable parameters that need to be initialized or established. The procedure followed to design the NN is described in depth in subsection 3.4., what lead us to obtain the best trained NN in terms of SCR improvement.

Once the best NN is achieved, some results obtained during the test stage are given. The results presented below consider the worst cases that compose the test set, i.e., the radar images where the worst performances are achieved. These worst cases refer to hard sea state conditions and strong radar measurements of the ship, where not only the radar cross section of the ship is elevated, the electromagnetic shadow of the ship is also of high intensity. The results exposed below present high levels of clutter reduction. But, as these results are obtained for the worst cases of the test set, it means that, in the remaining radar images of this set, the achieved clutter reduction is even higher than the one presented below. The reason why it happens is due to work with this kind of clutter and target conditions is difficult for the proposed system. Nevertheless, even for the worst cases mentioned above, the results are always satisfactory both in terms of subjective and objective measurements, as shown below. Moreover, the results obtained with the images selected for the training and validation sets are not shown because their results are even better than the ones obtained with the radar images of the test set, as could be observed from the average results shown in table 3.

**Figure 5.**
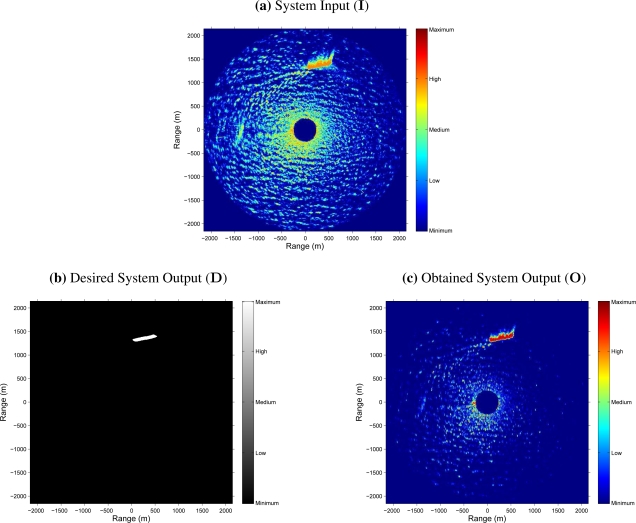
Example of Scan Maps containing Target and Clutter at the Input and Output of the NN-based Clutter Reduction System

**Figure 6.**
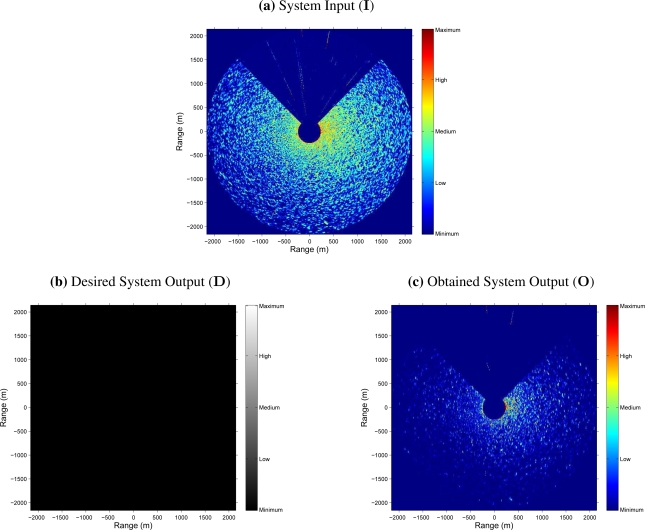
Example of Scan Maps containing only Clutter at the Input and Output of the NN-based Clutter Reduction System

As an example, Figure 5 shows the result obtained after an input radar image is processed by the NN-based clutter reduction system. As exposed above, the radar image considered for this example belongs to the test set. Moreover, it contains sea clutter (sea state level 3 following the criteria established by the WMO) and target (a ship of type *Cruise*) radar returns. In this case, it is observed that in the NN-based clutter reduction system output (subfigure (c) of Figure 5), the level of signal where clutter is present (dark zone in subfigure (b) of Figure 5) is decreased and the level of signal where target is present (clear zone in subfigure (b) of Figure 5) is increased with respect to the input (subfigure (a) of Figure 5). Nevertheless, the following questions could arise: If there is not any target in the radar scene, what does it happen? Does the clutter reduction remains constant, greater or lower? In order to answer this kind of questions, Figure 6 shows the radar images at the input and output of the NN-based clutter reduction system when no target and only sea clutter (sea state level 4 following the criteria established by the WMO) is present in the radar scene. Moreover, a certain sector of 90° is rejected from the radar coverage. In this case, the output clutter level is again reduced. So, with both examples, the robustness of the proposed system to reduce the clutter level when different sea states are considered is shown. Note that the proposed system and its performance is also robust against the presence or absence of target in the radar image and independent of the azimuthal radar coverage.

### 4.4. NN-based Clutter Reduction System: Objective Analysis

The results presented in the previous subsection show graphically how the clutter is reduced and the target is enhanced. But, this graphical representation makes sometimes the comparison of the results obtained with one radar image with others difficult. In this way, this comparison is not easy because it has a certain subjective behavior (personal interpretation of the radar images at the system output). For this reason, an objective evaluation is needed (see subsection 3.5.).

One possible evaluation is by the MS error. So, if this objective characterization is calculated by eq. (11), considering *M* = 1, and using the desired (**D**) and obtained (**O**) system outputs of the cases presented in Figure 5 and 6, MS errors of 8.3 · 10^−3^ and 8.7 · 10^−3^ are achieved, respectively. It is important to note that the radar images **D** and **O** are normalized by a factor of $\frac{1}{255}$ (inverse of the image pixel dynamic range) because the output range of the NN varies from 0 to 1. Moreover, note that this error measurement is a mean value and, as can be observed in both figures, the clutter reduction obtained near the radar site is lower than far away.

Although the previous evaluation can be used to evaluate the system performance, in radar signal processing, the measurement of the error is not enough and a different objective evaluation is needed. In this way, the estimation of the clutter and target (when it is present) powers and the difference between them expressed in logarithmic units, i.e., the SCR (dB), at the input and output of the NN-based clutter reduction system are given. So, table 4 contains these power and SCR estimations, and their corresponding improvements achieved by the proposed clutter reduction system. As can be observed, the clutter power is approximately reduced in 8.7 dB (negative improvement) and the target power (when target is present) is improved in 1.3 dB at the system output. So, an SCR improvement (difference between the *P*_t_ and *P*_c_ improvements when target is present) of 10 dB is achieved, approximately. Note that this SCR improvement is a minimum value and better results are obtained because of both selected radar images are the worst cases that compose the test set under study when target is present or absent. In this case, the worst cases refer to hard sea state conditions and strong radar measurements of the ship, where the electromagnetic shadow of the ship is of high intensity (see Figure 5).

As mentioned above, Figure 5 and 6, and table 4 show the results obtained for two different radar images of two different radar image sequences considered in the test set. But, lets make now a deep analysis of the average results obtained for the whole training, validation and test sets. In this way, the second row (size 5*/*6*/*1) of table 3 shows the achieved average *P*_c_ and *P*_t_ improvements and the corresponding average SCR improvements for each set. These average measurements show how the *P*_c_ improvement is near −11.5 dB (power reduction) for each set, approximately. Note that this average measurement is greater than the *P*_c_ improvement achieved for the cases of Figure 5 and 6 (8.7 dB, approximately), what means that better results than the ones presented in these figures can be found in the test set, what was previously mentioned. Moreover, as can be observed, the achieved average *P*_t_ improvement is near 1.3 dB in average, which is similar to the one obtained in the case under study of Figure 5. Finally, the achieved SCR improvement is near 12.5 dB, approximately, which is greater than the one obtained for the case under study of Figure 5 (10 dB, approximately) because of the same reasons previously exposed for the analysis of the results of the average *P*_c_ improvement. On the other hand, it is important to note that the average SCR improvement is not always the same as the difference between the average *P*_t_ and *P*_c_ improvements. It is due to the average *P*_t_ and SCR improvements are only calculated for the radar images of each set that contain target information, whereas the average *P*_c_ improvement is calculated for all the radar images of each set, where a target can be present or not.

After this general analysis of the achieved results, the reader can observe how, when the training and validation sets are considered, the results achieved are better than the ones obtained when the test set is used. This effect is due to the NN learns the specific conditions of the data presented in the training and validation sets during its training. But, as the NN will be working in certain conditions that are different of the design ones (test conditions), its performance will be slightly different (usually lower). Nevertheless, note that as the training and validation sets were composed in order to cover several working conditions of the system, the results achieved for the three sets are similar each other.

## 5. Conclusions and Future Research Lines

After the developed study of the proposed NN-based clutter reduction system, several conclusions (advantages) can be extracted and several future research lines are opened.

The first conclusion is related to the good performance achieved by the NN-based clutter reduction system for different environmental radar conditions (sea state) and situations (different kind of targets are present or not). This performance can be characterized according to a subjective criterion. This criterion is based on the observation of the radar images obtained at the output of the proposed clutter reduction system. But, due to the subjective interpretation of these results, another way to characterize its performance is proposed. This characterization is based on objective criteria. These criteria include measurements such as the MS error, the clutter power reduction and the target power enhancement, what lead us finally to propose the measurement of the SCR improvement as a final parameter to characterize the system when target is present. In this way, clutter power reductions greater than 11 dB and target power enhancements greater than 1 dB are achieved in average at the output of the proposed NN-based clutter reduction system, both in design and test stages. So, once the NN-based clutter reduction is designed, SCR improvements greater than 12 dB are achieved in average for radar images never seen before during the design stage of the system.

The second conclusion is related to the great robustness achieved by the proposed clutter reduction system against changes in the environmental conditions (different sea states). This robustness is supported by the similar performances achieved during the design and test stages, because of all the radar image sequences considered in both stages are different each other. Moreover, this robustness is warranted independently a target is present or not in the radar scene. Anyway, it is important to note that due to the sea clutter conditions varies with time, the system will warranty a minimum performance because of its above-mentioned robustness.

And the third one, which is focused on implementation purposes, is related to the low computational cost needed to implement the proposed clutter reduction system. In this way, once the NN is designed (trained), which is based on the proposed structure of size 5*/*6*/*1, a total of (5 · 6 + 5 · 1) + (6 · 1 + 1 · 1) = 43 memory cells to store the NN synaptic weights and biases are only needed. Moreover, a total of 5 · 6 + 6 · 1 = 36 products (the biases does not need a product), 5 · 6 + 6 · 1 = 36 additions and 6 + 1 = 7 evaluations of the neuron activation functions are needed to implement it. So, considering the time to access to memory/registers and the multiplication, addition and evaluation times, the processing time of the NN-based clutter reduction system is very low, what involves high speed radar image processing.

Finally, this paper does not try to set a closed NN-based clutter reduction system. It tries to open new research lines. In this way, and due to the low MLP size found to be optimum in the system, second order approaches (e.g. Newton, Quasi-Newton and the Levenberg-Marquardt optimization techniques [16]) to train NNs could be used to accelerate the convergence speed of the training algorithm and to reduce the time of system design. Moreover, as ANFIS architecture can give significant performance improvements on NNs and other techniques, such as fuzzy logic [13], this architecture could be used to enhance the clutter reduction achieved by the proposed system.

## Figures and Tables

**Table 1.** Radar Measurement System Characteristics in Transmission and Reception

Radar System Frequency (X-band)	10.0 GHz
Antenna Rotation Speed	50 rpm
Antenna Polarization	H and H
Pulse Repetition Frequency (PRF)	1000 Hz
Radar Pulse Width	80 ns
Distance Range (Coverage)	200 – 2150 m
Range Resolution	7.5 m
Azimuthal Range (Coverage)	0 – 360 °
Azimuthal Resolution	0.28 °

**Table 2.** Kind of ships considered in the study and their typical dimensions

*Kind of Ship*	*Length*	*Beam*
Cruise Ships	350 m	35 m
Ferries	200 m	28 m
Container Ships	200 m	32 m
General Cargo Ships	120 m	18 m

**Table 3.** Average Improvements of the Clutter Power (*P*_c_), Target Power (*P*_t_) and SCR during the Design and Test Stages in the *Training/Validation/Test Sets* for Different NN-based Clutter Reduction System (MLP) sizes (5*/H/*1)

MLP size (5*/H/*1)	Average *P*_c_ Improvement (dB)	Average *P*_t_ Improvement (dB)	Average SCR Improvement (dB)
5/4/1	−11.4 / −11.3 / −11.1	+1.2 / +1.2 / +0.9	+12.6 / +12.6 / +12.1
**5/6/1**	−11.7 / −11.5 / −11.3	+1.3 / +1.3 / +1.1	**+12.9 / +12.8 / +12.5**
5/8/1	−11.3 / −11.1 / −10.9	+1.3 / +1.3 / +1.2	+12.6 / +12.5 / +12.3
5/10/1	−11.2 / −11.1 / −10.9	+1.2 / +1.3 / +1.1	+12.4 / +12.3 / +12.0
5/12/1	−11.0 / −10.9 / −10.8	+1.3 / +1.3 / +1.1	+12.4 / +12.3 / +12.0
5/14/1	−11.0 / −10.9 / −10.8	+1.2 / +1.2 / +1.0	+12.4 / +12.3 / +12.0
5/16/1	−11.0 / −10.9 / −10.7	+1.2 / +1.2 / +1.0	+12.4 / +12.3 / +11.9
5/18/1	−10.9 / −10.8 / −10.7	+1.2 / +1.2 / +1.0	+12.2 / +12.1 / +11.9
5/20/1	−10.6 / −10.5 / −10.3	+1.2 / +1.2 / +0.9	+12.0 / +11.9 / +11.6
5/25/1	−10.5 / −10.4 / −10.2	+1.2 / +1.2 / +0.8	+12.0 / +11.9 / +11.4
5/30/1	−10.5 / −10.4 / −10.0	+1.2 / +1.2 / +0.7	+11.8 / +11.7 / +11.0

**Table 4.** Clutter Power (*P*_c_), Target Power (*P*_c_) and Signal-to-Clutter Ratio (SCR) Improvements for a NN-based Clutter Reduction System with a NN Structure of 5/6/1

	Radar Image	Input (dBm)	Output (dBm)	**Improvement (dB)**
Clutter Power (*P*_c_)	(Fig. 5)	+6.1	−2.7	**−8.8**
Target Power (*P*_t_)	(Fig. 5)	+15.5	+16.8	**+1.3**
Signal-to-Clutter Ratio (SCR)	(Fig. 5)	+9.4	+19.5	**+10.1**

Clutter Power (*P*_c_)	(Fig. 6)	+6.2	−2.4	**−8.6**
Target Power (*P*_t_)	(Fig. 6)	-	-	**-**
Signal-to-Clutter Ratio (SCR)	(Fig. 6)	-	-	**-**
